# Supplementary material for: Splice-Junction-Based Mapping of Alternative Isoforms in the Human Proteome
Source: Cell Rep. Author manuscript; Available in PMC 2020 Jan 15. (PMC6961840; doi:10.1016/j.celrep.2019.11.026)
Supplement: 2 [file NIHMS1546469-supplement-2.zip › DF1/PXD010154/Prostate/Prostate_1_SVIL_RPELCTSHSETPTVDDEEKVDER.pdf]

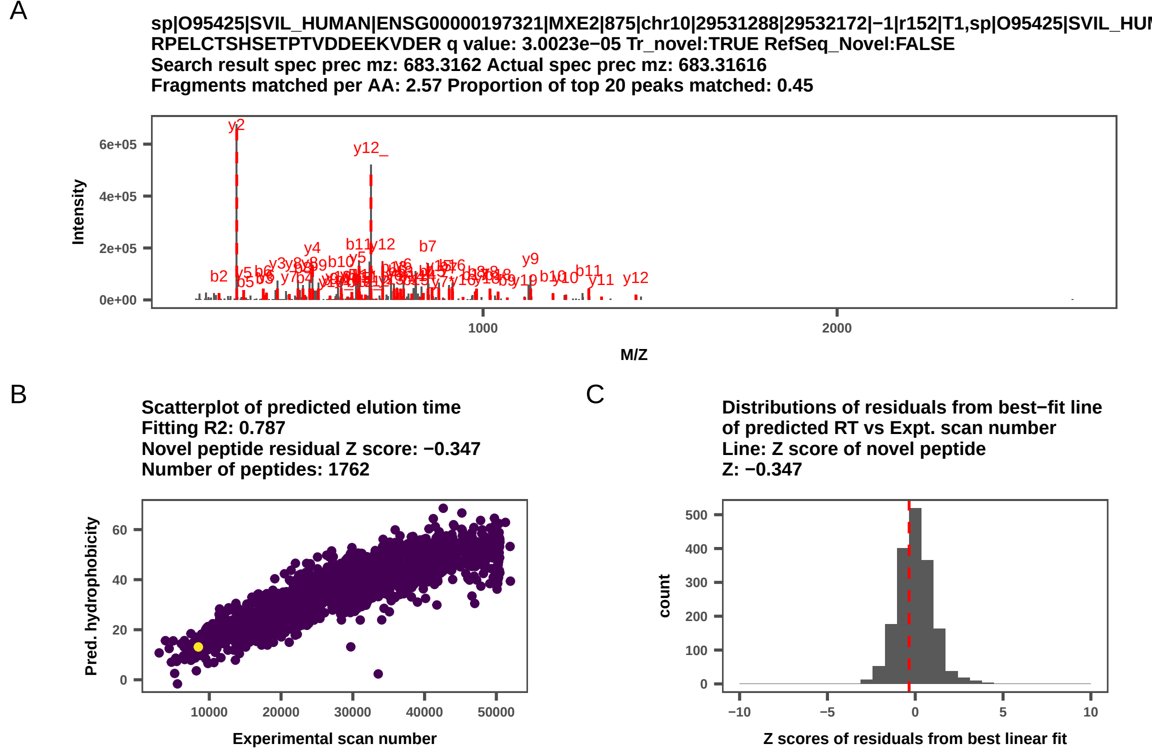
A
sp|O95425|SVIL_HUMAN|ENSG00000197321|MXE2|875|chr10|29531288|29532172|−1|r152|T1,sp|O95425|SVIL_HUM
RPELCTSHSETPTVDDEEKVDER q value: 3.0023e−05 Tr_novel:TRUE RefSeq_Novel:FALSE
Search result spec prec mz: 683.3162 Actual spec prec mz: 683.31616
Fragments matched per AA: 2.57 Proportion of top 20 peaks matched: 0.45
Intensity
6e+05
4e+05
2e+05
0e+00
y2
y12_
y4
b11
y12
b7
y9
b2
b10
b11
y11
y12
1000
2000
M/Z
B
Scatterplot of predicted elution time
Fitting R2: 0.787
Novel peptide residual Z score: −0.347
Number of peptides: 1762
Pred. hydrophobicity
60
40
20
0
10000
20000
30000
40000
50000
Experimental scan number
C
Distributions of residuals from best−fit line
of predicted RT vs Expt. scan number
Line: Z score of novel peptide
Z: −0.347
count
500
400
300
200
100
0
−10
−5
0
5
10
Z scores of residuals from best linear fit
